# Imaging-based spectrometer-less optofluidic biosensors based on dielectric metasurfaces for detecting extracellular vesicles

**DOI:** 10.1038/s41467-021-23257-y

**Published:** 2021-05-31

**Authors:** Yasaman Jahani, Eduardo R. Arvelo, Filiz Yesilkoy, Kirill Koshelev, Chiara Cianciaruso, Michele De Palma, Yuri Kivshar, Hatice Altug

**Affiliations:** 1 Institute of Bioengineering, École Polytechnique Fédérale de Lausanne (EPFL), Lausanne, Switzerland; 2 Department of Biomedical Engineering, University of Wisconsin–Madison, Madison, WI USA; 3 Nonlinear Physics Center, Research School of Physics, Australian National University, Canberra, Australia; 4 School of Physics and Engineering, ITMO University, St Petersburg, Russia; 5 Swiss Institute for Experimental Cancer Research (ISREC), School of Life Sciences, École Polytechnique Fédérale de Lausanne (EPFL), Lausanne, Switzerland

**Keywords:** Diagnostic markers, Biomedical engineering, Metamaterials, Imaging and sensing

## Abstract

Biosensors are indispensable tools for public, global, and personalized healthcare as they provide tests that can be used from early disease detection and treatment monitoring to preventing pandemics. We introduce single-wavelength imaging biosensors capable of reconstructing spectral shift information induced by biomarkers dynamically using an advanced data processing technique based on an optimal linear estimator. Our method achieves superior sensitivity without wavelength scanning or spectroscopy instruments. We engineered diatomic dielectric metasurfaces supporting bound states in the continuum that allows high-quality resonances with accessible near-fields by in-plane symmetry breaking. The large-area metasurface chips are configured as microarrays and integrated with microfluidics on an imaging platform for real-time detection of breast cancer extracellular vesicles encompassing exosomes. The optofluidic system has high sensing performance with nearly 70 1/RIU figure-of-merit enabling detection of on average 0.41 nanoparticle/µm^2^ and real-time measurements of extracellular vesicles binding from down to 204 femtomolar solutions. Our biosensors provide the robustness of spectrometric approaches while substituting complex instrumentation with a single-wavelength light source and a complementary-metal-oxide-semiconductor camera, paving the way toward miniaturized devices for point-of-care diagnostics.

## Abstract (web summary)

The authors engineer a type of bound states in the continuum in diatomic dielectric metasurfaces, allowing for high-quality resonances with accessible enhanced fields. Metasurface microarrays are integrated with microfluidics on an imaging platform for real-time detection of biosamples, based on reconstructing spectral shift information.

## Introduction

Reliable, rapid, and highly sensitive detection of disease indicators is crucial for timely and specific diagnostics and treatments^1–3^. Point-of-care (POC) devices are highly desirable for health monitoring and biosafety applications in the field settings as they offer easy-to-use, low-cost and fast detection of numerous indicative biomarkers, such as proteins, antibodies, nucleic acids, and extracellular vesicles, including exosomes, as well as pathogens such as viruses^4–7^. For instance, the 2020 coronavirus outbreak underlines the importance of POC tools that can enable rapid, cost-effective, accurate, and quantitative tests for COVID-19. Optical biosensors based on nanophotonics are eminently sought after as they allow miniaturized lab-on-a-chip technologies for label-free analysis and multiplexed sensing^4,5,8,9^. When integrated with microfluidics, nanophotonic biosensors can perform automated measurements on low sample volumes and provide quantitative and real-time results without requiring time-consuming and expensive external labels^10–13^. Nanophotonic resonators can enable label-free detection of biomarkers by means of refractometric sensing that exploit optical resonances to monitor the changes in the dielectric properties in the vicinity of the resonators caused by, for instance, the binding of analytes to the surface-immobilized capture molecules. This change in a local refractive index translates into a resonance wavelength shift, which can be employed to quantify analyte binding events. Spectral interrogation is the conventional method to precisely track the resonance wavelength and measure the spectral shift induced by the analyte presence employing commercial spectrometers. The spectroscopic detection has been implemented with numerous nanophotonic resonators, including plasmonic nanoaperture arrays, nanoparticles, and nanoantennas such as rods, tubes, disks, and bow ties^13–17^.

In recent years, there has been a surge of efforts to improve both performance and functionalities of nanophotonic biosensors employing new structures, materials, and devices^6,18,19^. While optimizing the nanostructures for refractometric sensing, one should take into account multiple parameters, including the quality factor (*Q*) of the resonators, the enhancement factor for the near-field intensities, and the spatial overlap of the enhanced fields with the target analytes. As these parameters tend to be coupled with each other, it is also essential to consider their involved trade-offs (see Supplementary Information note 1). For example, high *Q* resonances with sharp spectral features are desirable to resolve the spectral shift induced by analyte binding at a superior resolution. However, if the high *Q* values are achieved by confining the mode tightly inside the nanoresonators, then this can reduce the overlap of the near-fields with the analytes and lower the overall sensitivity. Plasmonic sensors have been taking advantage of large near-field enhancement factors, while dissipative losses in metals lead to low *Q* resonances, which limits their overall sensing performance^20,21^. Recently, high-index all-dielectric metasurfaces driven by the physics of optical Mie resonances have emerged as an alternative platform as they do not suffer from losses due to the absence of free charges^22–31^. Silicon-based metasurfaces operating within the visible and near-IR (600–900 nm) spectral ranges have been employed for bulk and thin-film sensing using spectrometers^32,33^. Concurrently, studies show that sharp spectral resonances can be achieved by using the concept of bound states in the continuum (BICs), in which the light wave at the resonance remains completely localized in the metasurface even though the state coexists with a continuous electromagnetic spectrum of the environment^34,35^. In practice, BICs are realized with high but finite values of the *Q* due to structural losses and imperfections and are usually termed “quasi BICs” or “supercavity modes”^36,37^. The BIC-inspired resonances in the symmetry-broken all-dielectric metasurfaces have recently been used for highly sensitive biosensors^38^. This earlier work interrogates dielectric metasurfaces supporting quasi-BIC modes using hyperspectral imaging towards high-performance biomolecule detection.

Although spectrometers and wavelength-scanning systems provide reliable spectral shift information for monitoring the changes in the optical response, they are bulky and expensive. In addition, spectrometers are limited in simultaneously collecting data from an array of sensors for implementing multiplexed bioassays. Wavelength-scanning systems can provide spatially resolved spectral information with hyperspectral imaging in a given field of view for monitoring signals from multiple sensing elements^38,39^. However, wavelength-scanning is cumbersome and time-consuming; hence, it cannot be used to gather time-resolved images for measuring molecular binding kinetics. Therefore, there is a need for simple and scalable optical read-out mechanisms that can extract both temporally- and spatially-resolved information from large sensor areas. To this end, red-shifting of the resonance spectrum by the binding of the analytes on the sensor surface can be quantified by tracking the changes in the intensity over a narrow spectral window instead of collecting spectrally resolved information over a broad bandwidth^8,40^. In particular, when combined with a small-scale light source – i.e., laser or light-emitting diode (LED) – and an imaging camera (i.e., complementary metal-oxide-semiconductor (CMOS) or charge-coupled device (CCD) cameras), the intensity-based optical readers can be ultra-compact, low-weight, and portable by eliminating the need for sophisticated spectroscopy instrumentations, including scanning and/or moving parts^41^. Thus, spectrometer-less readers can reduce cost and improve mechanical robustness^42^. Furthermore, by acquiring images over a large field-of-view, they can enable the implementation of microarrays for high-throughput screening applications^4^. For instance, microarrays can be used to detect multiple target analytes from a given sample in a single measurement, saving both time and precious biosamples^43^. Nonetheless, compared to spectral interrogation, intensity interrogation could not achieve sensitive and reliable detection as it is less tolerant of noise factors, such as inhomogeneties in sensors’optical responses and inadequate choice of the light source.

In this work, we introduce single-wavelength imaging-based nanophotonic biosensors for achieving superior sensitivity based on reconstructed spectral shift without cumbersome wavelength scanning and spectrometers. To achieve this goal, we employ quasi-BIC modes manifesting themselves through high-*Q* resonances of “diatomic metasurfaces” by generalizing and expanding the concept of BIC developed for asymmetric metasurfaces^37^. Diatomic metasurfaces are based on a dimer-type unit cell a.k.a “meta-molecule”, in which the dimer symmetry is broken by changing the ellipticity of one of the meta-atoms to support high *Q* resonance and strong field-analyte overlaps. Compared to single-unit metasurfaces, such diatomic structures provide advanced flexibility in the engineering of the in-plane asymmetry while keeping the design easier to fabricate and make it more feasible for biosensing applications. We combine these metasurfaces with advanced data processing techniques for aided imaging biosensing. Our biosensor acquires large-area intensity images of the metasurface in real-time by using a single wavelength illumination and processes them optimally via a linear estimation algorithm to reconstruct spectral shift data at high accuracy dynamically. For demonstration, we integrated antibody functionalized metasurface chips in a 2D microarray format with microfluidics and performed in-flow detection and real-time binding of breast cancer extracellular vesicles, which are important biomarkers for diagnostics owing to their connection with disease pathology^44,45^. Our optofluidic sensor has enabled detection of on average 0.41 nanoparticle/μm^2^ and real-time quantification of extracellular vesicles binding from solutions with as low as 1.23 × 10^8^ particles/mL, the equivalent of 204 femtomolar. By fusing the simplicity of intensity-based large-area and real-time imaging detection with the robustness of spectroscopic systems, our technique paves the way for reliable and ultra-sensitive POC devices powered by temporally and spatially resolved biosensing spectral shift information.

## Results

### The experimental platform

**Fig. 1:**
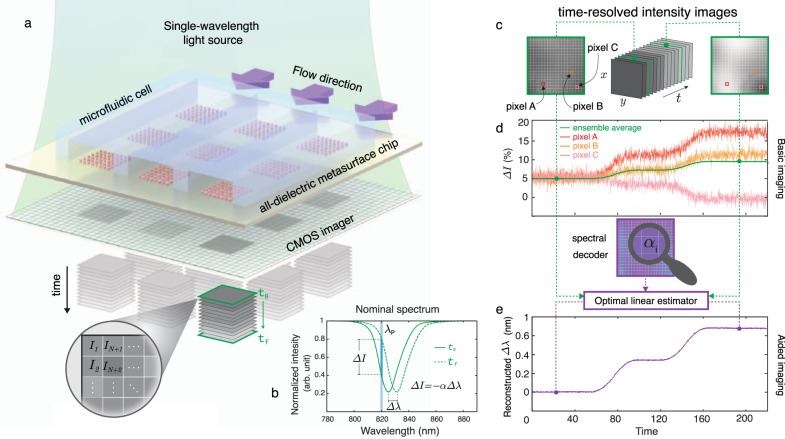
Principles of the single-wavelength algorithm-aided imaging biosensor with spectral shift reconstruction and its advantages over the basic imaging approach that merely relies on the intensity change. **a** Sketch of a real-time in-flow imaging platform showing a 2D microarray of all-dielectric sensors integrated with a microfluidic cell consisting of three independent flow channels. The metasurface chip is illuminated with a single-wavelength light source and imaged with a large-area CMOS camera to acquire intensity maps of the sensors in pixel resolution (*I*_1,_ *I*_2_, …, *I*_*N*+1_, *I*_*N*+2_,…). **b** Biomarker binding induces a red-shift in the transmission spectrum (Δ*λ*), which can be detected by tracking the resonance wavelength or by the intensity change (Δ*I*) at a fixed probing wavelength (*λ*_p_). Δ*I* can be approximated as linearly proportional to Δ*λ* with a constant of *α*, which is the slope of the transmission spectrum in the linear region near *λ*_*p*_. **c** Pictorial representation of time-resolved single-wavelength intensity images. **d** The illustration represents the shortcomings of the basic imaging approach. Time-resolved intensity data from single pixels (A, B, and C) from the sensor area give contradicting responses with noisy signals (red, orange, and pink curves) due to the nonuniformities. Ensemble averaging of the responses from multiple pixels can help to decrease the noise, although it may reduce the final signal, as depicted with the green curve. **e** The illustration represents the implementation and the advantages of our aided imaging approach. By leveraging the optimal linear estimator algorithm and the spectral decoder that incorporates the 2D map of the slope values, time-resolved single-wavelength intensity images are used to reconstruct robust spectral shift (Δ*λ*) information dynamically (purple curve).

Our all-dielectric biosensor, comprising a 2D microarray of metasurfaces, is assembled to a microfluidic unit containing three independent and simultaneously accessible flow channels made of Polydimethylsiloxane (PDMS). The optofluidic chip is illuminated with a single-wavelength light beam that excites the resonators. A CMOS camera acquires 2D images of the whole field of view at fixed time intervals to provide time-resolved intensity information from millions of CMOS pixels (Fig. 1a). Figure 1b shows the red-shift (Δλ) of the spectral response and its corresponding intensity change (ΔI) at a fixed probing wavelength (λ_p_) due to the binding of the analytes for each pixel. In contrast to spectral interrogation (i.e., measuring directly Δλ), basic imaging approaches only rely on the detection of the intensity changes. However, this makes basic imaging vulnerable to the noise factors such as the non-uniform optical response of the resonators over the sensor surface due to the fabrication imperfections and the inadequate choice of the illumination source (i.e., λ_*p*_). Figure 1d schematically illustrates the problems of the real-time intensity change data extracted from time-resolved images at different pixel locations. The signal coming from a single-pixel is not only noisy, but also contradicts the signals coming from the adjacent pixels (red, orange, and pink curves). Traditional practice to mitigate the noise is to ensemble average the signals from multiple pixels. However, this practice tends to reduce the final signal (green curve) and lowers the sensitivity for operation at low analyte concentrations. To address these shortcomings, we utilized an imaging method relying on the robust spectral shift (Δλ) information that is extracted without requiring bulky and expensive spectroscopy instrumentation. By using an optimal linear estimation algorithm and a spectral decoder that is unique to each metasurface sensor, we reconstruct spectral shift data from time-resolved single-wavelength intensity images dynamically. This algorithm-aided imaging approach can increase the signal to noise ratio (SNR) and, consequently, improve the limit of detection (LOD) by unveiling the signals coming from a highly diluted analyte, which is otherwise unresolvable by conventional ensemble averaging methods (Fig. 1e).

### All-dielectric metasurfaces

**Fig. 2:**
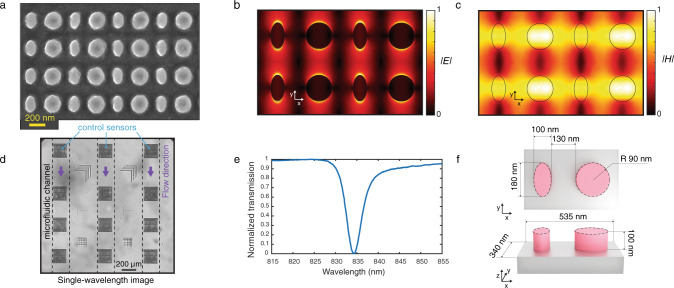
Diatomic dielectric metasurface supporting high quality factor quasi-BIC resonances. **a** Scanning electron microscopy (SEM) image of the fabricated dielectric metasurface. To prevent charging while taking SEM images, we applied a thin layer of Electra, a carbon-based conductive polymer. **b**, **c** Distributions of electric and magnetic fields, respectively, in four neighboring metaunits from numerical calculations. **d** A region of interest from an intensity image obtained at the resonance wavelength of the integrated optofluidic chip showing 3 × 4 all-dielectric metasurface sensors and borders of the three Polydimethylsiloxane (PDMS) flow channels. **e** Experimentally acquired transmission spectrum of the fabricated dielectric metasurfaces in y-polarization. **f** Top and side-views schematic of the metaunit composed of an elliptical (long and short axis 180 nm and 100 nm respectively) and a cylindrical (90 nm in radius) a-Si resonators.

To engineer our all-dielectric metasurfaces for biosensing, we developed the universal theory of quasi-BICs in broken-symmetry dimer-type metasurfaces (see Supplementary Information Note 2). The advantage of such diatomic metasurfaces is a higher number of degrees of freedom to achieve better precision in tuning the accessible enhanced near-fields and the quasi-BIC *Q*. The meta-molecule consists of a circular and an elliptic disk—this allows breaking the in-plane inversion symmetry (Fig. 2f). The asymmetry is defined as the degree of ellipticity of one of the meta-atoms, which is proportional to the difference between the ellipse long and short axes. We observed that controlling the asymmetry along one axis improves the tolerance to the fabrication imperfection than the alternative methods such as the one used in our previous work^38^. Nonzero ellipticity is a perturbation of a circular disk and induces leakage of radiation out of the metasurface plane at the BIC conditions (Supplementary Fig. 3). The BIC transforms into a quasi-BIC with finite radiative losses controlled by the asymmetry of the meta-molecule. Due to the reciprocity, the quasi-BIC manifests itself as a narrow resonant feature in the transmission spectrum for y-polarized incident light (Supplementary Fig. 3). Our design supports highly accessible enhanced electric and magnetic fields in the external surface of the dielectric nanoresonator (Fig. 2b, c). The local electric field enhancement factors can reach as high as almost 5000 times (Supplementary Fig. 4), and the enhancement of the electric field averaged over a volume of a 5-nm thick layer in the vicinity of the nanoresonators in an aqueous media is calculated to be 12.4 times. Overall, this design gives rise to a considerable intensification of the optical interaction with the analytes and makes the platform suitable for sensing applications^46–48^.

Figure 2a shows a scanning electron microscope (SEM) image of the resonators fabricated by nanostructuring a 100-nm-thick amorphous silicon (a-Si) on top of a fused silica substrate using a top–down fabrication method with CMOS compatible techniques. Silicon is chosen as the constituent material due to its biocompatibility, well-established surface biofunctionalization methods, and access to mature CMOS fabrication facilities. Also, silicon benefits from desirable optical properties, including a high refractive index and relatively low loss in the near-IR range below 850 nm. The metasurfaces operate in the aqueous media with their resonance in the visible and near-IR spectral range (600 nm to 850 nm). Working in this range offers several unique advantages, including the wide availability of high-performance and cost-effective silicon-based CMOS and CCD cameras. For fabrication, we used the design with geometrical parameters shown in Fig. 2f, which corresponds to the ellipticity of 0.44. As mentioned above, the metasurface design is tolerant to fabrication imperfections due to the chosen symmetry-breaking strategy. The fabricated resonators were characterized to have <2% size deviations over the sensor area. In turn, a good homogeneity is observed in the optical response of the resonators (see supplementary information note 4).

Our metasurface chip comprises twelve 250 µm by 250 µm sensors in a 3 × 4 microarray format. The optofluidics incorporates three independent microfluidic channels, which allow the sensors to function in aqueous media for in-flow measurements and preserve analyte biofunctionality. The field of view (FOV) of the setup is >7 mm^2^; thus, we can acquire the images of all of the twelve sensors on a chip in a single shot. Figure 2d shows a subset of an intensity image obtained at the resonance wavelength of the all-dielectric metasurface. The sensors, as well as the borders of the three PDMS fluidic channels, are visible at high contrast. The cross patterns in between the microfluidic channels are alignment and focus monitoring marks, which are used during the subsequent data processing of the time-resolved intensity images. Our aided imaging approach, based on an optimal linear estimation algorithm and a spectral decoder, allows for precise reconstruction of the spectral shift over large areas from the acquired images. The spatially resolved reconstructed shift data from a large FOV has a compelling advantage over the traditional spectrometers by allowing for a concurrent control test and multiplexing to detect a panel of biomarkers in parallel. Figure 2e shows the experimentally recorded transmission spectrum of the engineered sensor. The optical characterization of 12 sensors, which are sampled with more than 950,000 CMOS pixels in the field of view, shows that the full-width at half-maximum (FWHM) of the resonance is as low as 2.3 nm and on average 4.57 nm with a standard deviation of 0.47 nm. Consequently, a *Q* as high as 250 and on average 178.6 with a standard deviation of 15.8 was recorded experimentally. Owing to the high-*Q* resonances and highly accessible enhanced near-fields, we achieved an experimental bulk refractive index sensitivity of 305 nm/RIU and a figure-of-merit (defined as sensitivity divided by FWHM) of 68 1/RIU.

### Aided imaging method

**Fig. 3:**
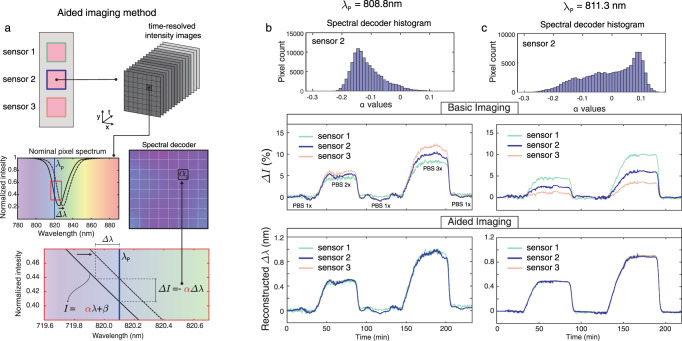
Implementation of the algorithm-aided imaging technique with reconstructed spectral shift information. **a** Time-resolved intensity data is fed to the optimal linear estimation algorithm with a spectral decoder, which is the two-dimensional (2D) map of the slope (*α*) of the transmission spectrum for each CMOS pixel at the probing wavelength (*λ*_p_). **b** At *λ*_p_ = 808.8 nm, the histogram of *α* values from a single sensor is populated mostly with negative values because, for the majority of the pixels, the resonance is probed at the left flank of the transmission dip (top panel). This case represents a sensor having a relatively homogenous optical response across its surface. For experimental bulk sensing, under a constant running of phosphate buffer saline (PBS) 1x (*n* = 1.33441), three sensors are exposed to a varying medium with PBS 2x (*n* = 1.33584) and PBS 3x (*n* = 1.33731) and their ensemble-averaged intensity changes (Δ*I*, middle panel) and reconstructed spectral shifts (Δ*λ*, bottom panel) are presented as a function of time. **c** At *λ*_p_ = 811.3 nm, the histogram of the same sensor has a similar number of positive and negative *α* values (top panel). This case represents a sensor having an inhomogenous optical response. For the same bulk sensing experiment used in Fig. 3b, the ensemble-averaged Δ*I* response of different sensors shows significantly varying signals (middle panel). In contrast, the reconstructed spectral shift data obtained by aided imaging (bottom panel) is consistent between the three sensors.

Figure 3 elucidates the gained robustness and tolerance of the biosensor when the aided imaging method is implemented compared to the basic imaging. To reconstruct spectral shift (Δλ), we fed the time-resolved intensity data to an optimal linear estimation algorithm that uses a “spectral decoder” and the recorded intensity change of each CMOS pixel to compute the best estimate of Δλ for the entire sensor. The spectral decoder of a sensor is built up by gathering the values of pixel-wise slopes (*α*) across the sensor area. To do so, we linearize the spectrum associated with each pixel around the probing wavelength λ_*p*_ and compute its slope (see Fig. 3a). This can be simply obtained by recording two images using narrowband illuminations: one at λ_*p*_ and a second one at λ_*p*_ + *δ*, for a small value of *δ*. This decoder provides information about the weight of the contribution of each pixel to the spectral shift so that the more sensitive ones have a higher influence on the estimation of Δ*λ*. In this work, the spectral decoder was computed using a hyperspectral imaging system where the sensor was imaged at wavelengths with a small spectral increment of 0.1 nm, and the extracted *α* values are mapped as a 2D matrix. To experimentally demonstrate the advantages of our aided imaging method over basic imaging, we studied two cases of uniform and non-uniform optical responses over the sensor area at different probing wavelengths. The experiments involved recording simultaneously time-resolved intensity images of three different sensors assembled in a microfluidic channel and comparing their responses under the same condition. This is achieved by flowing aqueous solutions with varying refractive indices across the sensors with consecutive injections of phosphate buffer saline (PBS) at different concentrations. Figure 3b, c show the responses of the three sensors at the illumination wavelengths of 808.8 nm and 811.3 nm, respectively. In the first case shown in Fig. 3b (top panel), the spectral decoder histogram of a representative sensor (sensor 2) is populated dominantly with negative values, indicating that the probing wavelength intersects the spectra of most pixels on the left flank of the resonance dip. Even with relatively uniform distributions, the responses of the three sensors obtained with basic imaging differ in the intensity change sensorgram (Fig. 3b, middle panel) compared to the aided imaging method (Fig. 3b, bottom panel). If the nonuniformity increases, the performance of the basic imaging deteriorates even more, as shown with the second case in Fig. 3c. In this figure (top panel), the spectral decoder histogram has a similar number of positive and negative values. Consequently, in the ensemble averaging with the absolute intensity change, the pixels with oppositive-valued slopes work against one another and result in significant variations between the three responses, leading to poor sensing performance (Fig. 3c, middle panel). In contrast, when the spectral decoder is used in the context of the optimal estimator, we obtained almost identical responses from all three sensors (Fig. 3c, bottom panel). The drastically improved performance illustrates that our technique is robust to the inhomogeneity of sensor response that is caused by, for instance, small fabrication imperfections or the improper choice of probing wavelength. This is a significant step toward inexpensive POC instruments for on-site use in resource-limited settings since it eliminates the need for the elusive perfect fabrication of the sensors as well as allows for flexibility in the choice of probing wavelengths. Moreover, our technique provides the reconstruction of the spectral shift without the need for a bulky and expensive spectrometer.

### Biosensing experiments

To demonstrate the biosensing functionality of our aided imaging method, we performed real-time in-flow detection of nanoparticles (NPs), as well as biological extracellular vesicles (EVs) secreted from cancer cells with an immunoassay. Initially, biotinylated silica nanoparticles (100 nm diameter) were used as the mimics of bioparticles (i.e., EVs and viruses) for the characterization of the sensors^49,50^. Each microfluidic flow channel interacts with four dielectric metasurface sensors (see Fig. 2d). The first sensor, which is blocked with bovine serum albumin (BSA), serves as a control sensor. The successive three sensors (detection sensors) are locally functionalized with streptavidin protein to target the biotin molecules on the silica nanoparticles (Supplementary Fig. 6). A steady flow of phosphate buffer saline (PBS) 1X delivers a sequence of different dilutions of the NP solution to the sensors, which are continuously illuminated with a linearly polarized single-wavelength light and imaged in time intervals of 14 s by a CMOS camera. The reported Δ*λ* in the *y*-axis is the reconstructed shift observed on the detection sensors from which the reconstructed shift of the control sensor is subtracted to account for any non-specific bindings.

**Fig. 4:**
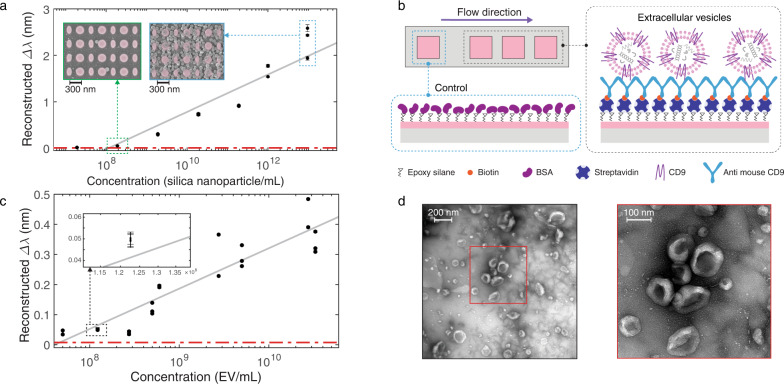
Biosensing using reconstructed spectral shift based on aided imaging method. **a** The reconstructed spectral shift (Δ*λ*) calibration curve of biotinylated silica nanoparticles (100 nm diameter). The insets are scanning electron microscopy images of the sensors with bound silica nanoparticles on resonators after introducing suspensions with 1.9 × 10^8^ nanoparticles/mL and 9.5 × 10^12^ nanoparticles/mL from left to right, respectively. The represented data points in the calibration curves (**a**, **c**) are the mean Δ*λ* over time from each sensor over a time-interval consisting of 81 consecutive images after the analyte binding reaches the equilibrium state, and the error bars indicate the standard deviations. The reconstructed Δ*λ* data points are obtained from seven dilutions of silica nanoparticle solutions over three independent sensors to achieve 21 data points on the calibration curve. The reconstructed Δ*λ* data from the detection sensors are corrected to the non-specific bindings on the control sensor. Some data points are overlapped due to the good agreement between the sensor performance. **b** Biorecognition assay to detect extracellular vesicles (EVs) on the detection sensors and on the control sensor blocked with bovine serum albumin (BSA) to account for non-specific binding is shown on top. **c** The reconstructed spectral shift calibration curve of EVs. The inset is the same plot with a magnified *x*-axis to better resolve the small error bars for an exemplary data point. The reconstructed shift data points from the detection sensors are corrected to the non-specific bindings on the control sensor. The represented data points are collected from nine different concentrations of EV solutions diluted from three independent batches measured on eight independent sensors to yield 24 data points on the calibration curve. Some data points are overlapped due to the good agreement between the sensors. **d** Transmission electron microscopy images of EVs from the cell culture supernatant of 4T1 mouse breast cancer cells can be seen at the bottom; the magnifications are ×23,000 and ×30,000 from left to right, respectively.

The calibration curve extracted from the reconstructed shift induced by the binding events of the silica nanoparticles on the all-dielectric metasurfaces is shown in Fig. 4a. The error bars in the figure are representing the standard deviation of Δ*λ* over time (sensorgram) from each sensor over a time-interval consisting of 81 consecutive images after the analyte binding reaches the equilibrium state. The relative standard deviation for the reproducibility of Δ*λ* in the working range of sensors is observed to be between 0.33% and 3.97%. LOD is the smallest concentration that can be detected with reasonable certainty for a biosensor^51^. The dashed red line in Fig. 4a shows the three times the averaged standard deviation, which was calculated by averaging the standard deviation of the sensorgram signals at equilibrium states over at least four time-intervals, each consisting of 81 consecutive images. The LOD of 1.2 × 10^8^ nanoparticles/mL, the equivalent of 199 femtomolar that corresponds to 126 ng/mL, was extracted from the intersection of the dashed red line and the calibration curve. The lowest detected quantity of 1.9 × 10^8^ nanoparticles/mL or 315 femtomolar, corresponding to 200 ng/mL, was measured and further investigated with SEM images revealing the equivalent detection of 0.41 nanoparticles/µm^2^ averaged over 78 SEM images and the total area of 687 µm^2^ at randomly chosen locations on the sensors with the standard deviation of 0.26 nanoparticles/µm^2^ and 0.34 nanoparticles/µm^2^ as the median. The insets of Fig. 4a shows representative SEM images from the solutions with 1.9 × 10^8^ nanoparticles/mL and 9.5 × 10^12^ nanoparticles/mL from left to right, respectively.

To demonstrate the competence of the proposed method with a biorecognition assay, we detected and quantified EVs purified from the cell culture supernatant of 4T1 mouse breast cancer cells^45^. EVs, which include exosomes and microvesicles, are nanoscale bioactive membrane particles secreted by virtually all types of cells^52^. There is strong evidence that EVs derived from breast cancer cells influence tumor progression^45^. EVs carry molecular information from parental cells; therefore, they can be considered as biomarkers for early-stage cancer detection and for minimally or non-invasive diagnostics^44^. A transmission electron microscopy (TEM) image of the EVs is shown in Fig. 4d, and their purification process and quantification are briefly explained in the methods section. EVs are highly enriched in tetraspanins^52^. The tetraspanins superfamily is a family of proteins with at least 33 distinct members in mammals; among them, CD9 is the best-studied transmembrane protein and a versatile marker that has been used for EV subtypes, including exosomes^53,54^. In order to capture the vesicles, we targeted CD9 and immobilized the corresponding biotinylated KMC8 anti-CD9 monoclonal antibody on the detection sensors functionalized with streptavidin. The control sensor was blocked with BSA (Fig. 4b). As in the nanoparticle experiment, a pressure pump maintains the constant buffer (PBS 1x) flow in the microfluidic channel, and a syringe pump provides successive injections of EV samples in various concentrations. Figure 4c shows the calibration curve that uses the reconstructed shifts derived from single-wavelength real-time in-flow experiments to detect EVs from 4T1 mouse breast cancer cells. The error bars in the figure are calculated in the same manner as in Fig. 4a. In this case, the relative standard deviation for reproducibility varies between 0.50% and 8.11%. Our sensors have a limit of detection of 8 × 10^7^ EV/mL or 133 femtomolar, according to the intersection of the three times the averaged standard deviation (the dashed red line in Fig. 4c) with the calibration curve, and the lowest detected quantity was measured as 1.23 × 10^8^ EV/mL or 204 femtomolar. The EV protein content at these concentration values corresponds to 267 ng/mL and 423 ng/mL, respectively^45^. While typically correlating with the tumor burden, the average values of cancer-related EVs range between 10^9^ and 10^10^ per mL of human plasma^55–57^. With a LOD of 8 × 10^7^ EV/mL and about five orders of magnitudes dynamic range, our results indicate that the performance of the proposed sensor is clinically relevant for the detection of cancer-related EVs.

## Discussion

We demonstrated a label-free nanophotonic biosensor, which leverages an aided imaging approach based on novel data processing and an optofluidic chip comprising of all-dielectric metasurfaces. Our method enables the extraction of spectral shift information from single-wavelength intensity images at high accuracy while preserving temporal and spatial resolution. Importantly, it is based on simple imaging optics with a fixed-wavelength illumination and a CMOS camera to collect data over a large field of view in real-time. Compared to the basic intensity imaging readers that merely rely on intensity change, our approach aided by optimal linear estimation algorithm provides robustness to the spectral inhomogeneities across the sensor surface that can originate from fabrication imperfections and inadequate operation wavelength. The current device, which images over 7 mm^2^ field-of-view, can be used to perform simultaneous measurement from hundreds of individual sensors in a microarray format. Hence our approach is suitable for multiplexed detection of a panel of biomarkers in-real time for high-throughput monitoring. Our diatomic all-dielectric metasurface, emanated from quasi-BIC modes, enables high-performance sensing by supporting high *Q* resonances with accessible near-fields. Furthermore, its unique symmetry-breaking design makes it more tolerant of nanofabrication. Using CMOS-compatible techniques, we fabricated highly uniform silicon metasurfaces operating below 850 nm in aqueous media and integrated with microfluidics for in-flow measurements. As a proof-of-concept, we applied the new optofluidic device for real-time detection of breast cancer derived EVs, and by functionalization of the metasurfaces with specific antibodies, we were able to report the LOD of down to 133 femtomolar.

As an outlook, the flexibility of the detection principle can enable the expansion into various different application areas. For instance, with proper surface functionalization, we can selectively target other bioparticles such as intact viruses for infectious disease applications and smaller biomolecules, including proteins, antibodies, and nucleic acids. We anticipate wafer-scale and high-throughput fabrication techniques to eliminate the dependence on high-resolution e-beam lithography. This can drastically reduce the cost and enable disposable biochips customized as consumables for detecting different analytes. We foresee device integration in miniaturized biosensors by using small-scale light sources with tailored bandwidth and low-cost on-chip imagers. The use of large-area imaging data gives the opportunity of implementing other advanced computation techniques (i.e., artificial intelligence methods) towards smarter sensing. By eliminating the need for bulky and expensive instrumentation, the spectrometer-less device architecture can lead to affordable, compact, easy-to-use, and reliable point-of-care systems for disease diagnostics, personalized medicine, and democratization of health care in resource-limited settings.

## Methods

### All-dielectric metasurface fabrication

A thin layer of amorphous silicon (a-Si) with 100 nm thickness was deposited using low-pressure chemical vapor deposition (LPCVD) on a clean bare fused-silica wafer. The wafer was patterned using 100 KeV electron-beam lithography (EBL). First, the wafers were cleaned with radio corporation of America (RCA) cleaning solution and oxygen plasma. A positive-tone resist, poly(methyl methacrylate) in a double layer configuration (PMMA 495 K bottom and 950 K top) was spin-coated and baked for 5 min at 180 °C to yield the total thickness of 160 nm. PMMA double-layer configuration is crucial to make undercut structures to facilitate lift-off. A 10-nm gold layer is sputtered on the PMMA layer to ensure conductivity during electron-beam writing. After EBL exposure, the gold layer was etched away in KI + I_2_ solution, and the wafer was developed with a mixture of Methyl isobutyl ketone (MIBK) and IPA(Isopropyl alcohol) in 1–3 ratios. To make a hard mask, a 30-nm-thick aluminum layer was deposited using an electron-beam evaporator followed by dicing the wafer into 1.5 cm × 1.5 cm chips. Metal lift-off was done by soaking the chips in a resist-remover bath at 70 °C for 6 h. The fabrication process concludes with transferring the nanostructures to the a-Si layer using fluorine-based deep reactive ion etching, and finally, hard mask removal in aluminum etch solution.

### Optical characterization setup

All-dielectric metasurfaces were optically characterized using a hyperspectral (HSI) platform. The HSI system consists of a supercontinuum laser (SuperK EXTREME EXR-15, NKT photonics) coupled to a tunable filter (LLTF, NKT photonics) integrated with an inverted microscope (Eclipse-Ti, Nikon). The collimated linearly polarized laser line (1.75 nm bandwidth at 700 nm) light was transmitted from the metasurface and collected with a ×10 objective, conveyed through a collinear analyzer and imaged with a CMOS camera (DS Qi 2, Nikon). To extract the transmission spectrum of all-dielectric metasurfaces with pixel size spatial resolution over a large field of view, a hyperspectral data cube was acquired by continuously imaging the chip synchronized with the sweep of the illumination source wavelength. The hyperspectral data cube of a chip was normalized to the hyperspectral data cube of the light source. The normalized intensity profile of each pixel over the wavelength sweep yields the transmission spectra of that pixel. The devices were connected and controlled with a centralized Matlab code.

### Single -wavelength imaging-based real-time biosensing measurements

The optical setup for the real-time in-flow measurements was the same as the optical characterization setup. A biofunctionalized chip was assembled to a microfluidic structure and placed on a microscope stage. The microfluidic system consists of a pressure pump (Elveflow, OB1) to maintain the constant buffer (PBS 1x) flow in the microfluidic channel, a syringe pump (Advanced MicroFluidics, LSPone) for analyte injection with a flowmeter (Elveflow, FS3) and tubing. Our data was collected from the net sample volume of 150 µL, which is more than one order of magnitude smaller than a conventional blood test. This volume can be further decreased to few tens of µLs if in-flow measurements and real-time data are not needed, and the measurements are performed under static conditions. Image acquisition in 14-second intervals and data pre-processing were handled with the centralized Matlab code assisted by a macro from NIS Nikon software. The pre-processing module was capable of providing live feedback by plotting the reconstructed spectral shift over time.

### Full-wave numerical analysis

Numerical analysis of the all-dielectric metasurfaces was performed using a commercially available finite-element frequency-domain solver (CST Microwave Studio 2018).

### Microfluidic structure fabrication

The microfluidic structures were made by soft lithography using a silicon master mold to achieve three 300 µm wide individual flow channels that are simultaneously accessible. The master mold was made with photolithography techniques to achieve the patterns of the final microfluidic channel. A film of positive-tone resist (AZ 1512, 1.3 µm) was coated and patterned on a silicon wafer using an automatic coater and developer (EVG 150, EV Group) and direct laser writer (MLA 150, Heidelberg). The patterned photoresist was used as an etching mask for fluorine-based silicon dry etch in a deep reactive ion etching (DRIE) tool (AMS 200, Adixen) to make 25 µm thick patterns. Finally, the photoresist is removed with oxygen plasma cleaning. One mold can be used repeatedly if the surface is protected from permanent adhesion of PDMS by silanization. Silanization was done using TriMethylChloroSilane (TMCS) in a desiccator under vacuum to make TMCS evaporate and form a passivation layer on the mold surface. The base and crosslinker precursors were mixed in a 10 to 1 ratio to make PDMS. The precursor mixture was degassed in a desiccator and poured into the mold. After another degassing step to ensure removing all the bubbles from the mold’s surface, it was baked at 80 °C for 4 h. Finally, the inverted replica of the mold in PDMS was peeled off from the mold, and each pattern was cut, punched, and bound to its complementary part with oxygen plasma.

### Surface chemistry

Covalent surface chemistry based on (3-glycidoxypropyl) trimethoxysilane (3-GPS) (Sigma-Aldrich) monolayer was used to immobilize the capture molecules on the all-dielectric metasurface. The silane end bonds to the silicon oxide and covers the all-dielectric metasurface with a uniform 3-GPS monolayer. The epoxide functional group of 3-GPS binds to the amine group of the capture molecules. First, the chips were cleaned in RCA solution at 50 °C for 30 min. The clean chips were incubated in the 3-GPS solution in toluene (1% vol/vol) for 20 min and rinsed in fresh toluene to remove the unreacted 3-GPS molecules and dried. The chips were baked for 30 min at 120 °C and were stored under a vacuum to be used within a week.

### Biotinylated silica nanoparticles biorecognition assay

To specifically target the Biotinylated silica nanoparticles (Creative Diagnostics) of 100 nm diameter, streptavidin was covalently immobilized on the detection sensors as a capture molecule, and BSA (Sigma-Aldrich) was immobilized on the control sensor. Epoxy-silane chemistry described above is used for the immobilization of a monolayer of Streptavidin and BSA molecules on the detection and control sensors, respectively. 40 droplets (480 pL per droplet) of Streptavidin (Thermo Scientific, 1 mg/mL) and BSA (1% wt/vol) in PBS (Sigma-Aldrich) 1x buffer were spotted on the sensors with a low-volume liquid dispenser (sciFLEXARRAYER S3, Scienion). The spotted chip is incubated for 16 hours at 4 °C.

### EV biorecognition assay

All-dielectric metasurfaces were initially treated with epoxy-silane chemistry and functionalized with a monolayer of streptavidin on the detection sensors and BSA on the control sensors by spotting technique. Streptavidin and BSA molecules are incubated for 2 h at room temperature and rinsed consecutively with PBS 1x and Mili-Q water, and then dried. In all, 40 droplets (480 pL per droplet) of a biotinylated capturing molecule, KMC8 monoclonal antibody (Biotin Rat Anti-Mouse CD9, BD biosciences, 0.5 mg/mL) in PBS 1x were spotted on detection sensors and BSA on the control sensors. The spotted chip is incubated for 16 h at 4 °C.

#### EV isolation

EVs were purified from media conditioned by 4T1 cells. In all, 4T1 cells (mammary adenocarcinoma; from ATCC) were cultured in 15 cm tissue culture-treated dishes in Iscove’s Modified Dulbecco’s Medium (IMDM, Sigma) supplemented with 10% fetal bovine serum (FBS; Gibco), l-glutamine (Amimed), and penicillin/streptomycin (Gibco). Cells at ~40–50% confluence were moved to a medium containing 5% EV-depleted FBS^45^. EVs were isolated from the conditioned medium using sequential ultracentrifugation (500 × *g* for 5 min; 2000 × *g* for 10 min; 4600 × *g* for 20 min; 134,000 × *g* for 70 min). The resulting EV pellet was washed in PBS, ultracentrifuged again at 134,000 × *g* for 70 min, and finally dissolved in PBS, which was obtained by ultracentrifugation of standard FBS at 134,000 × *g* for 16 h at 4 °C followed by filtration through a 0.1-mm vacuum filtration bottle. After 3 days, the conditioned medium was collected and centrifuged at 500 × *g* for 5 min, 2000 × *g* for 10 min to remove dead cells and debris, and ultracentrifuged at 4600×*g* for 20 min at 4 °C to remove large vesicles. The supernatant was then transferred to new tubes and ultracentrifuged at 134,000 × *g* for 70 min at 4 °C to collect small EVs. The EV pellet was then washed in 35 mL of PBS and ultracentrifuged again at 134,000 × *g* for 70 min at 4 °C. All ultracentrifugation steps were performed using a Beckman ultracentrifuge and a SW32Ti rotor. The resulting EV preparation was resuspended in PBS and stored at −80 °C.

### Transmission electron microscopy of purified EVs

Purified EVs (5 µg in 15 µL of PBS) were applied to carbon-coated 400 mesh grids (Electron Microscopy Sciences) for 5 min, then washed with PBS and stained with 2% uranyl acetate (Sigma) for 30 s. Excess stain was removed by touching the very edge of the mesh with a piece of filter paper (Whatman). Grids were then let dry completely prior to acquisition. Images were obtained using a transmission electron microscope device (Tecnai Spirit, FEI Company).

### Computation of spectral decoder

Given a probing wavelength λ_p_, 11 images are recorded at wavelengths according to the column vector *λ* = [*λ*_p_−0.5, …, *λ*_p_,…, *λ*_p_ + 0.5] nm. For each pixel *i*, the recorded intensities are normalized according to the intensity value corresponding to *λ*_p_, resulting in a vector *y*^*i*^, where *y*^*i*^[6] = 1. The slope associated with pixel *i* is then given by Eq. (1)

1 $${\alpha }^{i}=\frac{{({y}^{i}\mbox{-}\overline{{y}^{i}})}^{T}({\lambda }^{i}\mbox{-}\overline{{\lambda }^{i}})}{{({\lambda }^{i}\mbox{-}\overline{{\lambda }^{i}})}^{T}({\lambda }^{i}\mbox{-}\overline{{\lambda }^{i}})}$$

where the bar represents the mean value of the vector. Note that, while we use 11 images to improve the estimation of the slope, two images are sufficient. The computation of the spectral decoder was carried out in Matlab 2019b.

### Optimal linear estimator

Images acquired over time at the probing wavelength *λ*_p_ are first normalized to the average image over 10 acquisitions to establish a baseline. An image pixel *i* in a sensor area with *N* pixels has an associated (normalized) intensity value at time k given by *y*^*i*^_*k*_. The optimal linear estimator of the resonance shift at time *k* over the sensor area is given by Eq. (2).

2 $${\widehat{\varDelta \lambda }}_{k}=\frac{1}{{\sum }_{N}{(\alpha^i)}^{2}}{\sum }_{N}\alpha^i(1-{y}_{k}^{i})$$

For the utilized metasurfaces, the optimal linear estimator works well in the neighborhood of *λ*_*p*_, which ranges from 2 to 3 nm, depending on the spectral location of *λ*_*p*_. For values outside the linear regime, we implemented a correction factor to enable the measurement of large spectral shifts (i.e., silica nanoparticles at high concentrations). All computations were carried out in Matlab 2019b.

### Reporting summary

Further information on research design is available in the Nature Research Reporting Summary linked to this article.

## Supplementary information

Supplementary Information

Reporting Summary

## Data Availability

The data that support the plots within this paper and other findings of this study are available within the article, its supplementary information, or from the corresponding author upon reasonable request.

## Code availability

The custom codes used in this study are available from the corresponding author upon reasonable request.
