# Supplementary material for: Imaging-based spectrometer-less optofluidic biosensors based on dielectric metasurfaces for detecting extracellular vesicles
Source: Nat Commun. 2021 May 31;12:3246. doi: 10.1038/s41467-021-23257-y (PMC8167130; doi:10.1038/s41467-021-23257-y)
Supplement: Supplementary file 1 — Supplementary Information [file 41467_2021_23257_MOESM1_ESM.pdf]

Supplementary Information for

Imaging-based spectrometer-less optofluidic biosensors based on dielectric metasurfaces for detecting extracellular vesicles

Yasaman Jahani[1*], Eduardo R. Arvelo[1*], Filiz Yesilkoy[2], Kirill Koshelev[3,4], Chiara Cianciaruso[5], Michele De Palma[5], Yuri Kivshar[3], and Hatice Altug[1**]

[1]Institute of Bioengineering, École Polytechnique Fédérale de Lausanne (EPFL) 1015 Lausanne, Switzerland

[2]Department of Biomedical Engineering, University of Wisconsin–Madison, Madison, WI 53706, USA

[3]Nonlinear Physics Centre, Research School of Physics, Australian National University, Canberra ACT 2601, Australia

[4]Department of Physics and Engineering, ITMO University, St Petersburg 197101, Russia

[5]Swiss Institute for Experimental Cancer Research (ISREC), School of Life Sciences, École Polytechnique Fédérale de Lausanne (EPFL), Lausanne, 1015, Switzerland

**correspondence to Prof. Hatice Altug: hatice.altug@epfl.ch

*Equally contributed authors

Supplementary Figures:

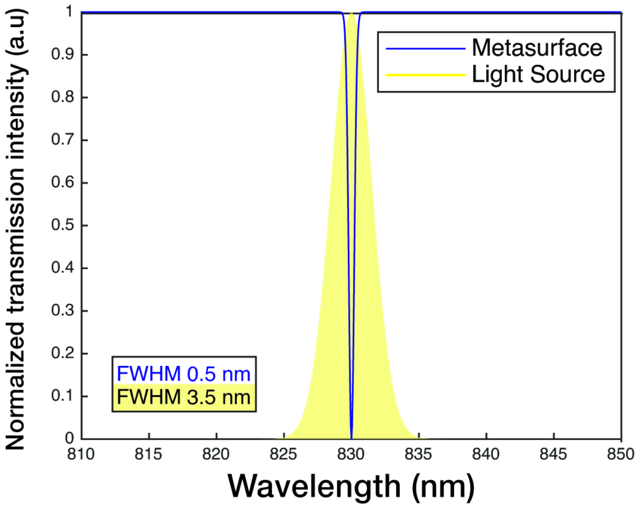

Supplementary Figure 1: Very sharp resonances cannot be resolved using a light source with a broader spectrum.

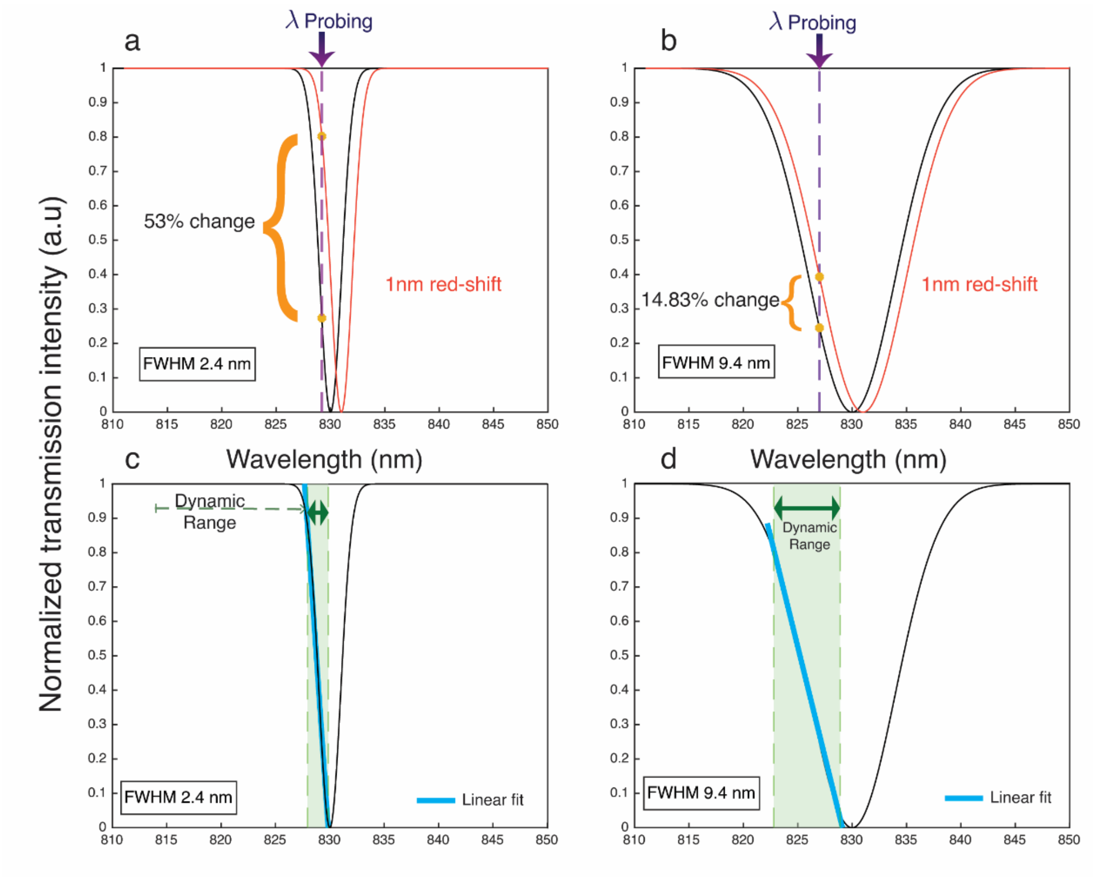

Supplementary Figure 2: The trade-offs involved in using higher and lower quality factor (Q) metasurfaces for sensing are showed graphically with representative curves. The narrowband illumination is chosen as a line (at a wavelength of λ_p probe) for a better illustration of the trade-offs. a) and b) show the influence of the Q value on the obtained intensity changes from the red-shifting of the resonance due to the analyte binding. a) shows that a higher Q resonance (346) with the full-width at half-maximum (FWHM) of 2.4 nm exhibits a 53% intensity change when the resonance is red-shifted for 1 nm. On the other hand, b) shows that a lower Q resonance (88) with the FWHM of 9.4 nm exhibits only a 14.83% intensity change for the same red-shift of 1 nm. c) and d) show the influence of the Q on the dynamic range of the sensor. Here, the dynamic range is chosen as the part of the resonance when the slope has less than 1% of deviations from the linear

fit. c) shows that as the higher Q resonance has smaller bandwidth, it leads to a smaller dynamic range. On the other hand, d) shows that a lower *Q* resonance offers a larger dynamic range.

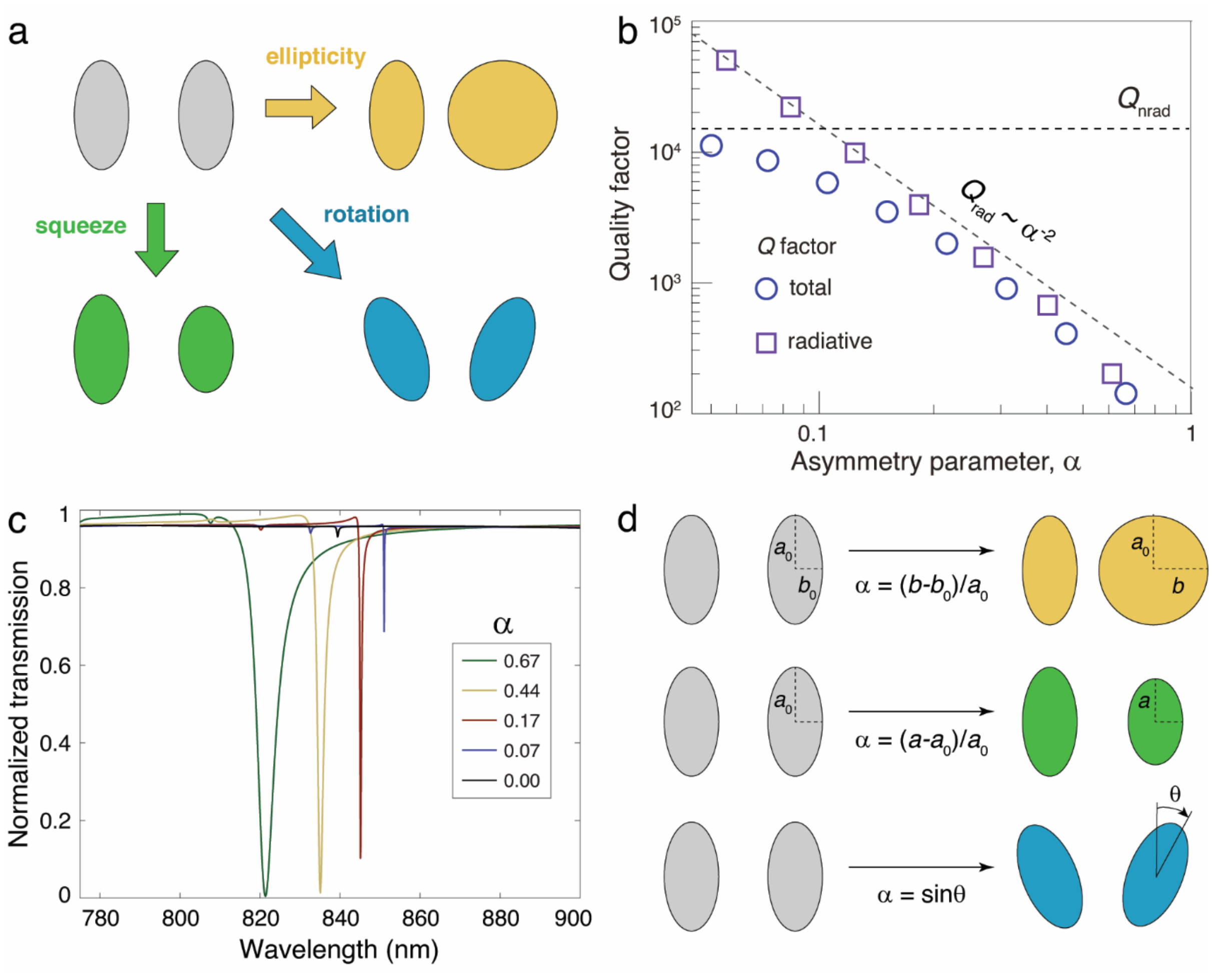

Supplementary Figure 3. BICs in diatomic metasurfaces. a) Different ways to introduce asymmetry for a diatomic metasurface: ellipticity, squeezing, rotation. b) Dependence of the quasi-BIC quality factor (*Q*) on the asymmetry parameter of the meta-molecule. Purple squares and blue circles show the radiative and total *Q*, respectively. The black dashed lines show a fit to the inverse square law and the value of the non-radiative *Q*. c) Characteristic evolution of the transmission spectra by changing the meta-molecule asymmetry. d) Definition of the asymmetry parameter via ellipticity, squeezing, and rotation for the same diatomic metasurface.

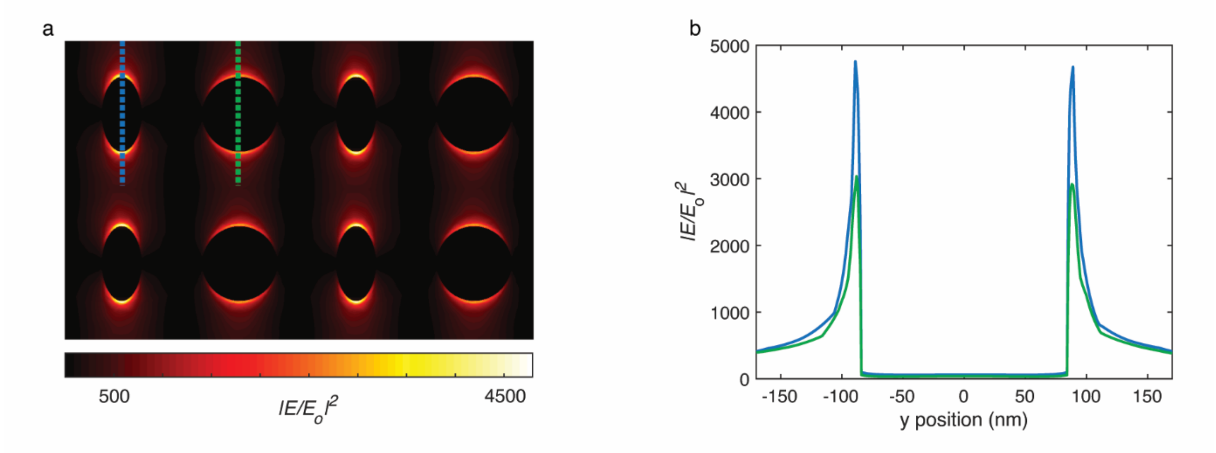

Supplementary Figure 4. Electric field enhancement in the vicinity of the resonators a) Electric field enhancement demonstrated for a cluster of four neighboring unit cells. b) Electric field enhancement along the y-axis of the elliptical (blue) and circular (green) disks.

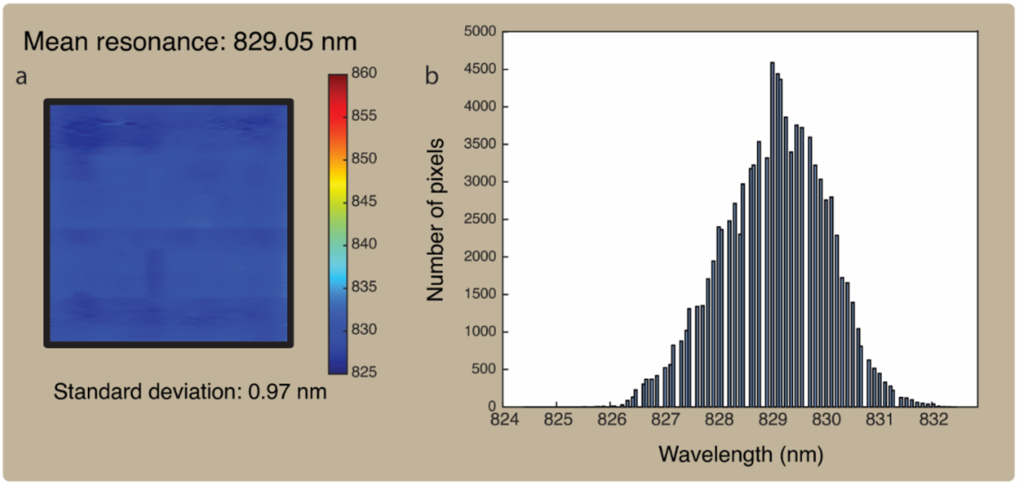

Supplementary Figure 5 Spectral characterization of the nanofabricated metasurfaces using hyperspectral imaging set-up across the sensor surface. a) The extracted resonance map and b) histogram of the resonance wavelength distribution of a fabricated sensor indicate that the mean resonance wavelength of the sensor is 829.05 nm with a standard deviation of 0.97 nm.

Supplementary Figure 6 Biorecognition assay to detect silica nanoparticles on the detection

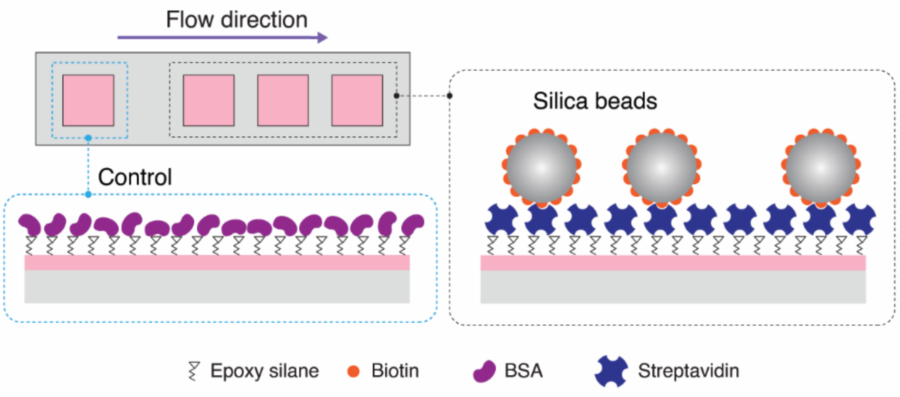

sensors and on the control sensor to account for non-specific binding. The control sensor is blocked with bovine serum albumin (BSA), while the detection sensors are functionalized with streptavidin.

Supplementary Note 1: Important parameters in designing refractometric biosensors

Multiple parameters affect the performance of a refractometric biosensor; hence, it is essential to consider the relevant parameters simultaneously. Below, we discuss some of them :

i) The quality factor (*Q*) contributes to the performance of a biosensor utilizing optical resonances. An ultra-high *Q* resonance resembling a delta function is not suitable for refractometric imaging-based detection because the intensity changes induced by the wavelength shifts cannot be used to resolve continuous variation of intensity (Supplementary Figure 1). On the one hand, it is preferable to have relatively sharp resonances, i.e., with steep slopes of the intensity profile, to provide bigger changes in the output signal for a given resonance shift. Supplementary Figure 2a and S2b show realistic cases with a high *Q* value of 346 and a lower *Q* of 88. A bigger output value leads to the easier detection of more dilute samples, resulting in a better limit-of-detection (LOD). On the other hand, a lower *Q* offers a wider dynamic range of a biosensor, as shown in the example (Supplementary Figures 2c and 2d). Thus, there is a trade-off between the LOD and dynamic range that we can achieve for a given biosensor.

ii) Another important consideration in designing metasurfaces for sensing applications is the enhancement of the near-fields and their accessibly to the analytes. It is essential that enhanced near-fields extend out of the resonator and spatially overlap with the samples on the sensor surface (see also Section 3). This also brings a trade-off because maximizing the field's spatial overlap with analytes (e.g., by not confining the mode tightly in a high-index meta-unit) reduces the Q value. Here, it is useful to emphasize that BIC designs that can achieve impressively high *Q*s with tightly confined field enhancements inside the resonators are less suited for biosensing and more appropriate for nonlinear or lasing applications.

iii) In biosensing applications, it is important to show operation in an aqueous environment because water is essential to preserve the bioactivity and extend its applicability for real-time and in-flow measurements. However, the optical loss of water hampers the high-Q values of the nanoresonators. In our work, we optimized a design for operation in solution (with the integration of a microfluidic chip) while still supporting suitably high Q value as well as accessible high near-field enhancements at 850 nm wavelength range.

Supplementary Note 2: The general concept of quasi-BICs in asymmetric diatomic metasurfaces

In this section, we develop a general theory of quasi-bound states in the continuum (quasi-BICs) in dielectric diatomic metasurfaces. Contrary to earlier works, here, we intentionally use a complex unit cell (meta-molecule) with two meta-atoms per unit. Compared to single-atom metasurfaces, diatomic structures provide advanced flexibility in the engineering of the in-plane asymmetry while keeping the design easy to fabricate and feasible for biosensing applications.

We consider a silicon metasurface composed of a square lattice of elliptical dimers deposited on a glass substrate. Parameters: disk height is 100 nm, x-period and y-period are 535 and 340 nm, respectively, the distance between disk centers is 270 nm, the radius of the short and long semi-axis of the ellipse is 50 nm and 90 nm, respectively. The structure supports a true BIC with an infinite quality factor at a wavelength of about 855 nm. To achieve a finite Q of the resonance, we transform the BIC to a quasi-BIC by introducing the in-plane asymmetry of the unit cell. Due to the reciprocity, the quasi-BIC can also be observed in response to an external excitation of the structure. For such diatomic metasurface, the asymmetry can be introduced in different ways, including squeezing of one meta-atom, changing its ellipticity, and rotation of one or two meta-atoms, as shown in Supplementary Figure 3a. The asymmetry parameter α is a linear function of perturbation and has a specific definition for each considered case, as shown in Supplementary Figure 3d. An important difference of diatomic metasurfaces from monoatomic is a higher number of degrees of freedom, which allows achieving better precision in the control of the quasi-BIC Q. Depending on the application and the device requirements, one can use the most suitable of the mentioned types of asymmetry to obtain a quasi-BIC with a specific Q and field enhancement. We

also note that, unlike squeezing and rotation, to the best of our knowledge, changing the ellipticity was not explored earlier as a way to introduce asymmetry for diatomic metasurfaces.

The eigenmode calculations show that for diatomic metasurfaces, the radiative Q of the quasi-BIC decreases with the increase of asymmetry following the inverse square law $Q_{\mathrm{rad}} = Q_0\alpha^{-2}$, as shown in Supplementary Figure 3b. For simulations of the radiative Q, we use the material dispersion for silicon without absorption losses. The total Q factor is limited by the non-radiative quality factor Q_{nrad}, which takes into account the material losses, fabrication imperfections, and sample finite $Q^{-1} = Q_{\mathrm{rad}}^{-1} + Q_{\mathrm{nrad}}^{-1}$. Assuming the weak dependence of Q_{nrad} on the asymmetry, we obtain a closed-form expression for the total Q (equation (1))

$$Q = \frac{Q_{nrad}}{1+\alpha^2 Q_{nrad}/Q_0} \quad (1)$$

which dependence on α is shown with blue circles in Supplementary Figure 3b.

In the transmission spectrum, the quasi-BIC produces a resonant dip with a Fano lineshape, which width and position depend on the asymmetry parameter. Characteristic normalized transmission spectra are shown in Supplementary Figure 3c. For $\alpha = 0$, sharp features in the transmission disappear, and the resonant mode becomes dark, so it does not manifest itself in the scattering spectra.

For the experimental design, we change the disk ellipticity increasing the smaller semi-axis of one of the meta-atoms to 90 nm, making it circular. Such perturbation corresponds to the ellipticity $\alpha = 0.44$. For the designed structure, the quasi-BIC mode is strongly confined to the surface of the resonators, making the metasurface an ideal platform for biosensing applications.

Supplementary Note 3: Numerical calculations of electric field enhancement factors

Electric field enhancement distribution over 4 unit cells and along the y-axis of each meta-atom are shown in Supplementary Figure 4.

Supplementary Figure 4b shows that the local electric field enhancement factors can reach as high as almost 5000 in the close proximity of the elliptical resonators. Moreover, numerical calculations of the enhancement of the electric field averaged over a volume of a 5 nm thick layer in the nanoresonators' vicinity indicate an enhancement of 12.4 times for the averaged field. The significant augmentation of the fields greatly enhances the optical interactions with the analytes making this design compelling for sensing applications.

Supplementary Note 4: Characterization of the fabricated resonators over the sensor area

In order to characterize the homogeneity of the nanofabricated resonators over the sensor, we performed image processing on over 100 SEM images. The statistics on over 800 resonators revealed the average size of the disk diameter as 182.72 nm with a standard deviation of 1.38 nm (0.75%) and the average size and standard deviation of the long and short axes of the ellipse as 178.25 nm ±3.17 nm (1.78%) and 104.93 nm ±1.72 nm (1.64%), respectively. The probing locations were chosen randomly all around the sensor and on the very edges and the corners. The deviation of less than 2% all around the sensor illustrates the high precision of our optimized nanofabrication procedure.

To spectrally characterize the uniformity of the structures, we used our hyperspectral imaging set-up[1]. This set-up enables the extraction of resonance maps, which gives the resonance wavelength over the entire sensor area at a single-pixel resolution. Supplementary Figures 5a and 5b show the

spectral resonance map and the histogram of resonance wavelength distribution for our metasurface, respectively.

The extracted mean value indicates a negligible difference between the simulation (835 nm) and the experiment (829.05 nm). The measurements also reveal a standard deviation of 0.97 nm, highlighting the uniformity in the optical response over the sensor area and the correspondingly high quality of the optimized nanofabrication process.

Supplementary Note 5: Bioregconitions assay for nanoparticles

To demonstrate the biosensing capability of the platform, we performed real-time in-flow detection of nanoparticles to mimic the bioparticles (i.e., extracellular vesicles and viruses). To target the biotinylated silica nanoparticles, streptavidin was immobilized on the detection sensors while the control sensor was blocked with bovine serum albumin (Supplementary Figure 6).

Supplementary References:

1. Yesilkoy, F. *et al.* Ultrasensitive hyperspectral imaging and biodetection enabled by dielectric metasurfaces. *Nat. Photonics* **13**, 390–396 (2019).
